# Comparison of In-Hospital Mortality and Clinical Outcomes Between Patients Aged More Than and Less Than 80 Years Undergoing Transcatheter Aortic Valve Replacement

**DOI:** 10.7759/cureus.24534

**Published:** 2022-04-27

**Authors:** Mukunthan Murthi, Sujitha Velagapudi, Bharosa Sharma, Olisa Ezegwu, Emmanuel Akuna, Dae Yong Park, Ramtej Atluri, Ufuk Vardar

**Affiliations:** 1 Internal Medicine, John H. Stroger, Jr. Hospital of Cook County, Chicago, USA

**Keywords:** pacemaker insertion, mortality, elderly, transcatheter aortic valve replacement, aortic stenosis

## Abstract

Background

The transcatheter aortic valve replacement (TAVR) procedure has been increasingly utilized in the management of aortic stenosis among the elderly. In this study, we sought to assess the hospital outcomes and major adverse events (MAEs) associated with TAVR in patients aged ≥80 years compared to those aged <80 years.

Methodology

We performed a retrospective observational study using the National Inpatient Sample in 2018. We divided TAVR patients into two cohorts based on age, namely, ≥80 years old and <80 years old. The primary outcomes included the comparison of in-hospital mortality and MAEs in the two cohorts.

Results

We identified 63,630 patients who underwent TAVR from January 1 to December 31, 2018. Among them, 35,115 (55%) were ≥80 years and 28,515 (45%) were <80 years of age. There was a higher rate of post-procedural in-hospital mortality in patients ≥80 years old (1.6% vs. 1.1%, adjusted odds ratio (aOR) = 1.56, [confidence interval (CI) = 1.13-2.16], p = 0.006). They also had higher rates of pacemaker insertion compared to those <80 years old (7.4% vs. 6.5%, aOR = 1.17 [CI = 1-1.35], p = 0.03). On subgroup analysis, the rates of MAEs were not different between the two cohorts (23.8% vs. 23.4%, p = 0.09); however, patients aged ≥80 years who experienced MAEs had higher in-hospital mortality (5.7% vs. 4.3%, aOR = 1.58 [CI = 1.08-2.32], p = 0.01) and shorter length of hospital stay (7.2 vs. 8.7 days, p = 0.03) compared to those aged <80 years. Anemia, liver disease, chronic kidney disease, and previous stroke were associated with higher odds of in-hospital MAEs in both groups.

Conclusions

The results of our study show that patients older than 80 years of age undergoing TAVR had higher rates of in-hospital mortality and pacemaker insertion compared to those less than 80 years of age. The rates of MAEs were not significantly different between the two groups.

## Introduction

Aortic stenosis is a common valvular disorder, especially among the elderly. The most common causes of aortic stenosis include bicuspid valve, calcification, and rheumatic heart disease [1]. About 5% of the population over 65 years of age have aortic stenosis and the percentage increases exponentially with older age. In addition, the prevalence of severe aortic stenosis increases with age, with 3.4-4.3% of adults over 75 years of age having severe aortic stenosis [2,3].

For many years, surgical aortic valve replacement (SAVR) has been the standard of care for severe symptomatic aortic stenosis until the advent of transcatheter aortic valve replacement (TAVR). TAVR is the alternative treatment option for patients considered unsuitable for surgery [4]. From the initial approval for patients with severe aortic stenosis and prohibitive operative risk, it is currently utilized even for severe aortic stenosis and low-risk patients [5,6].

Several studies have compared the outcomes of TAVR and SAVR. Some studies have also compared these outcomes specifically in octogenarians [7,8] and nonagenarians [9]. The results of these studies favor TAVR in patients older than 80 years which is reflected in the recent American Heart Association/American College of Cardiology guidelines [10]. However, there are limited data comparing the difference in outcomes of TAVR between patients aged more than 80 years and less than 80 years using large population databases. Therefore, in this study, we aimed to analyze the demographic characteristics and in-hospital outcomes after the TAVR procedure in patients ≥80 years old compared to those <80 years old. We also sought to investigate the factors that were independently associated with major adverse events (MAEs) in these two groups.

This article was previously posted to the medRxiv preprint server on February 23, 2022.

## Materials and methods

Study design and data source

We performed a retrospective study involving adult hospitalizations for the TAVR procedure in the United States by extracting data from the National Inpatient Sample (NIS) for the year 2018. The NIS is the largest publicly available all-payer inpatient admission database in the United States. It was developed by the Agency for Healthcare Research and Quality (AHRQ), Healthcare Cost and Utilization Project (HCUP), State Inpatient Databases (SID). This dataset includes discharge information for over seven million discharges annually with data stratified as a weighted sample. Discharge weights were calculated using post-stratification on hospital characteristics (census region, urban/rural location, teaching status, bed size, and hospital control) and patient characteristics (sex and five age groups: 0, 1-17, 18-44, 45-64, and 65 and older). Because the NIS does not include individual patient identifiers, this study did not require approval from the Cook County Health Institutional Review Board. This manuscript conforms with the Strengthening the Reporting of Observational Studies in Epidemiology (STROBE) statement for observational studies.

Study population and variables

We identified patients who underwent the TAVR procedure in 2018 using the International Classification of Diseases, Tenth Revision, Clinical Modification (ICD‐10‐CM) procedure codes. We further divided these patients into two groups based on their age, namely, ≥80 years old (group A) and <80 years old (group B). The NIS dataset includes variables on patient demographics, including age, gender, race, median household income, and type of insurance. It also contains hospital-level data, including hospital bed size, teaching status, and location. Comorbidities were identified using ICD-10 codes as well as Sundararajan’s adaptation of the modified Deyo’s Charlson comorbidity index (CCI) [11].

Measures of outcome

The primary outcomes were the comparison of in-hospital mortality and MAEs in patients who underwent the TAVR procedure stratified according to age. We included post-procedural hemorrhage, cardiac complications (acute myocardial infarction, heart failure, cardiac arrest, pericardial effusion, heart blocks, tachyarrhythmia, and bradyarrhythmia), acute kidney injury (AKI), stroke, and transient ischemic attack (TIA) as MAEs. Secondary outcomes included pacemaker insertion rate, the mean length of hospital stay (LOS), total hospital charges (THC), and independent predictors of MAEs.

Statistical analysis

Data were analyzed using Stata® version 16 software (StataCorp, College Station, TX, USA). We conducted all the analyses using the weighted samples for national estimates in accordance with HCUP guidelines. We calculated comorbidities as proportions of the cohorts and used the Chi-square test for comparison. We used univariate regression to identify variables affecting the primary outcomes. We included variables with a p-value of <0.1 in the final multivariate regression model. Variables identified to be significant by a literature review were also entered into the model. Subsequently, we performed a multivariate cox regression analysis to identify independent predictors of MAEs with p-values of <0.05 set as the threshold for statistical significance. For MAE analysis in all TAVR patients, the variables included age ≥80 years, gender, race, zip-code-wise median household income, region of hospital, insurance status, CCI category, chronic obstructive pulmonary disease (COPD), previous stroke, obesity, diabetes mellitus, peripheral vascular disease (PVD), liver disease, smoking history, and anemia. For MAE analysis in patients aged ≥80 years, the variables used in the multivariate analysis included age, gender, race, zip-code-wise median household income, region of hospital, insurance status, CCI category, COPD, previous stroke, obesity, diabetes mellitus, PVD, liver disease, smoking history, and anemia. For MAE analysis in patients aged <80 years, variables for multivariate analysis included age, gender, race, CCI category, insurance status, COPD, previous stroke, hypertension, diabetes mellitus, heart failure, chronic kidney disease (glomerular filtration rate of <60 mL/minute), liver disease, smoking history, and anemia.

## Results

**Figure 1:**
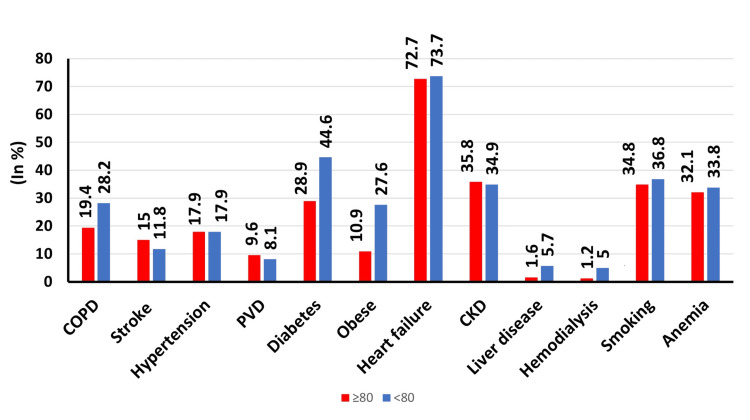
Graph showing the prevalence of comorbidities among TAVR patients aged ≥80 years versus <80 years. COPD: chronic obstructive pulmonary disease; PVD: peripheral vascular disease; CKD: chronic kidney disease; TAVR: transcatheter aortic valve replacement

We identified 63,630 patients who underwent TAVR from January 1 to December 31, 2018. Among them, 35,115 (55%) were in group A (mean age = 85, standard error (SE) = ±0.04] and 28,515 (45%) were in group B (mean age = 71, SE = ±0.11). Among group A and B patients, the proportion of females was 48% and 44%, respectively (p = 0.0002). Regarding ethnicity, European whites formed the major proportion of both groups. The number of patients with ≥three comorbidities was higher in group B (51.2% vs. 59.6%, p < 0.001). A larger proportion of patients in group B were privately insured compared to those in group A (3.8% vs. 12%, p > 0.001). All other baseline characteristics are presented in Table 1 and Figure 1.

**Table 1:** Clinical and demographic characteristics of TAVR patients. COPD: chronic obstructive pulmonary disease; PVD: peripheral vascular disease; CKD: chronic kidney disease; TAVR: transcatheter aortic valve replacement

Patient characteristics	≥80	<80	P-value
Subjects (n, %)	35,115 (55%)	28,515 (44%)	
Mean age (in years) (SE)	85 ± 0.04	71 ± 0.11	<0.001
Female (%)	48	44	0.0002
Race (%)			<0.001
White	88	83	
Black	3	6.2	
Hispanic	5	6.7	
Other	4	4	
Charlson category (number of comorbidities) (%)			<0.001
0	7.4	5	
1	20.6	15.6	
2	20.6	19.6	
≥3	51.2	59.6	
Zip-code-wise median income (in $) (%)			<0.001
1–45,999	18.3	24.7	
46,000–58,999	25.2	25.9	
59,000–78,999	27.6	26.7	
79,000+	28.8	23.1	
Insurance (%)			<0.001
Medicare	95.5	84	
Medicaid	0.3	3	
Private	3.8	12	
Other	0.1	0.7	
Hospital region (%)			<0.001
Northeast	23.2	19.1	
Midwest	22	22.4	
South	32.6	38	
Hospital bed-size (%)			0.17
Small	6.9	7.6	
Medium	21	19.5	
Large	72	72.8	
Location/teaching status of hospital (%)			0.51
Rural	0.8	0.6	
Urban non-teaching	9.1	8.8	
Urban teaching	90	90.5	
Comorbidities (%)			
COPD	19.4	28.2	<0.001
Stroke	15	11.8	<0.001
Hypertension	17.9	17.9	0.97
PVD	9.6	8.1	0.003
Diabetes	28.9	44.6	<0.001
Obesity	10.9	27.6	<0.001
Heart failure	72.7	73.7	0.25
CKD	35.8	34.9	0.32
Liver disease	1.6	5.7	<0.001
Hemodialysis	1.2	5	<0.001
Smoking	34.8	36.8	0.02
Anemia	32.1	33.8	0.04

Comparison of TAVR patients aged ≥80 years versus <80 years

**Figure 2:**
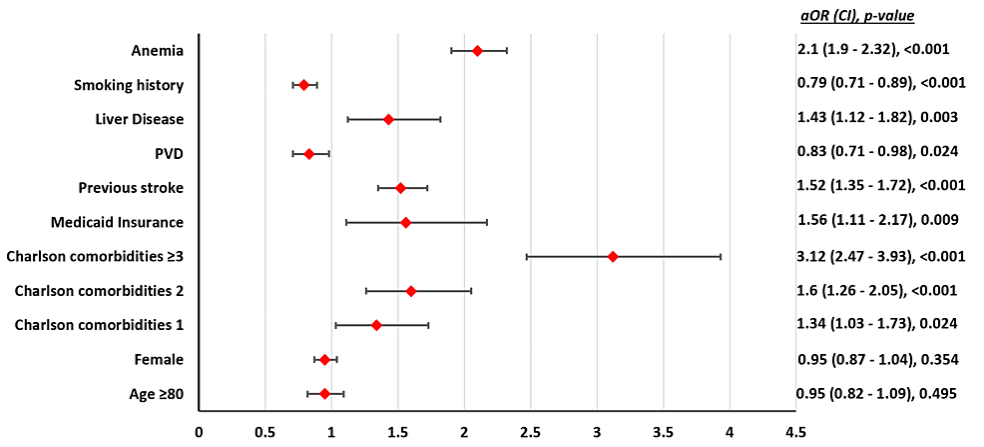
Independent factors associated with MAEs in patients undergoing TAVR. PVD: peripheral vascular disease; aOR = adjusted odds ratio; CI = confidence interval; MAE: major adverse event; TAVR: transcatheter aortic valve replacement

The in-hospital mortality rate for patients in group A and group B was 1.6% and 1.1%, respectively (adjusted odds ratio (aOR) = 1.56, [confidence interval (CI) = 1.13-2.16], p = 0.006). The rates of MAE were not different (23.8 vs. 23.4, p = 0.09). There were no significant differences in LOS (3.7 vs. 4.2 days, p = 0.26) and total hospital charges ($214,919 vs. $220,681, p = 0.42) between group A and group B (Table 2). Group A had higher rates of pacemaker insertion (7.4 vs. 6.5%, aOR = 1.17 [CI = 1-1.35], p = 0.03). On multivariate regression analysis, age ≥80 years was not independently associated with increased MAEs in TAVR patients. Figure 2 shows the independent factors associated with MAE.

**Table 2:** In-hospital outcomes and MAEs in TAVR patients stratified by age. ^#^Multivariate analysis. MAE: major adverse event; TIA: transient ischemic attack; AKI: acute kidney injury; TAVR: transcatheter aortic valve replacement

Variable (%)	≥80 years old	<80 years old	P-value^#^
MAEs	23.8	23.4	0.09
Post-procedural hemorrhage	1.7	1.7	0.54
Cardiac complications	13.2	12.9	0.51
AKI	10.3	11.3	0.14
Stroke and TIA	4.2	3.5	0.26
Pacemaker insertion	7.4	6.5	0.03
Died	570 (1.6%)	335 (1.1%)	0.006
Length of stay (in days)	3.7 (CI = 3.5-3.8)	4.2 (CI = 4-4.4)	0.26
Total hospital charges (in $)	214,919	220,681	0.42

Comparison of TAVR patients aged ≥80 years with and without MAEs

**Figure 3:**
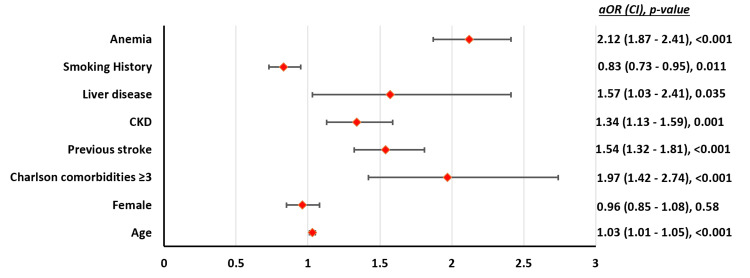
Independent factors associated with MAEs in patients aged ≥80 years undergoing TAVR. CKD: chronic kidney disease; aOR = adjusted odds ratio; CI = confidence interval; MAE: major adverse event; TAVR: transcatheter aortic valve replacement

The mean age for patients with and without MAEs was 85.5 (SE = ±0.08) versus 85.1 (SE = ±0.04), respectively (p < 0.001). Patients with MAEs had significantly higher comorbidities compared to those without MAEs (Table 3). Anemia (aOR = 2.12), liver disease (aOR = 1.57), CKD (aOR = 1.34), history of stroke (aOR = 1.54), and a higher number of comorbidities (aOR = 1.97) were independently associated with higher odds of MAEs (Figure 3). Increasing age was also associated with worse outcomes (aOR = 1.03).

**Table 3:** Comparison of patients stratified according to age based on the presence of major adverse events. ^*^P-value comparing patients aged ≥80 and <80 years with MAEs; ^#^Multivariate analysis MAE: major adverse events; COPD: chronic obstructive pulmonary disease; PVD: peripheral vascular disease; CKD: chronic kidney disease; TAVR: transcatheter aortic valve replacement

Patient characteristics	≥80 with MAEs	≥80 without MAEs	P-value	<80 with MAEs	<80 without MAEs	P-value	P-value*
Mean age (in years) (SE)	85.5 ± 0.08	85.1 ± 0.04	<0.001	71.2 ± 0.19	71.4 ± 0.13	<0.001	<0.001
Female (%)	47.3	48.1	0.55	44.6	44.7	0.92	0.13
Race (%)							
White	87.6	88.2	0.31	82.1	83.4	0.10	0.01
Black	3.3	3.1		6.2	6.3		
Hispanic	6.1	4.8		1.9	6.1		
Other	2.7	3.1		3.3	3.7		
Charlson category (number of comorbidities) (%)							
0	3.9	8.5	<0.001	1.9	6	<0.001	<0.001
1	13.5	22.9		8.6	17.8		
2	15.4	22.2		14.8	21.1		
≥3	67	46.2		74.5	55		
Zip-code-wise median income (in $) (%)							
1–45,999	17.7	18.5	0.46	23.1	24.4	0.68	<0.001
46,000–58,999	24	25.5		27	25.6		
59,000–78,999	28.4	27.3		26.7	26.7		
79,000+	29.7	28.5		23	23.2		
Comorbidities (%)							
COPD	21.8	18.7	0.006	31.7	27.1	0.0006	<0.001
Stroke	19.8	13.4	<0.001	15.9	10.6	<0.001	0.008
Hypertension	12.3	19.6	<0.001	12.1	19.7	<0.001	0.87
PVD	9.8	9.5	0.81	8.8	7.9	0.28	0.37
Diabetes	33.9	27.3	<0.001	48.9	43.2	0.0003	<0.001
Obese	11.1	10.9	0.81	27.4	27.7	0.81	<0.001
Heart failure	78.6	70.9	<0.001	79.5	71.9	<0.001	0.55
CKD	49.6	31.5	<0.001	50.6	30.1	<0.001	0.59
Liver disease	2.6	1.3	0.0002	8.5	4.8	<0.001	<0.001
Hemodialysis	1.7	1	0.01	4.2	5.2	0.15	<0.001
Smoking	32.9	35.4	0.09	32.4	38.1	0.0002	0.77
Anemia	48.2	27	<0.001	49.3	29.1	<0.001	0.55
Insurance (%)							
Medicare	95.8	95.4	0.42	82.8	84.5	0.008	<0.001
Medicaid	0.2	0.4		4.4	2.6		
Private	3.6	3.9		11.9	12.1		
Other	0.3	0.1		0.6	0.7		
Hospital region (%)							
Northeast	24.3	22.9	0.76	18.9	19.2	0.98	0.004
Midwest	22.3	22		22.1	22.5		
South	32	32		38.4	37.8		
West	21.2	22.1		20.4	20.3		
Hospital bed size (%)							
Small	6.6	6.9	0.93	6.4	8	0.08	0.19
Medium	20.9	20.9		18.1	19.8		
Large	72.3	72		75.4	72.1		
Location/teaching status of hospital (%)							
Rural							
Urban non-teaching	0.7	0.8	0.88	0.2	0.7	0.06	0.07
Urban teaching	9	9.1		8.1	9		
	90.2	90		91.6	90.2		
Outcomes							
Died (%)	5.7	0.3	<0.001^#^	4.3	0.2	<0.001^#^	0.01^#^
Length of stay (in days)	7.2	2.6	<0.001^#^	8.7	2.8	<0.001^#^	0.03^#^
Total charges ($)	283,618	193,473	<0.001^#^	300,624	196,224	<0.001^#^	0.17^#^

Comparison of TAVR patients aged <80 years with and without MAEs

**Figure 4:**
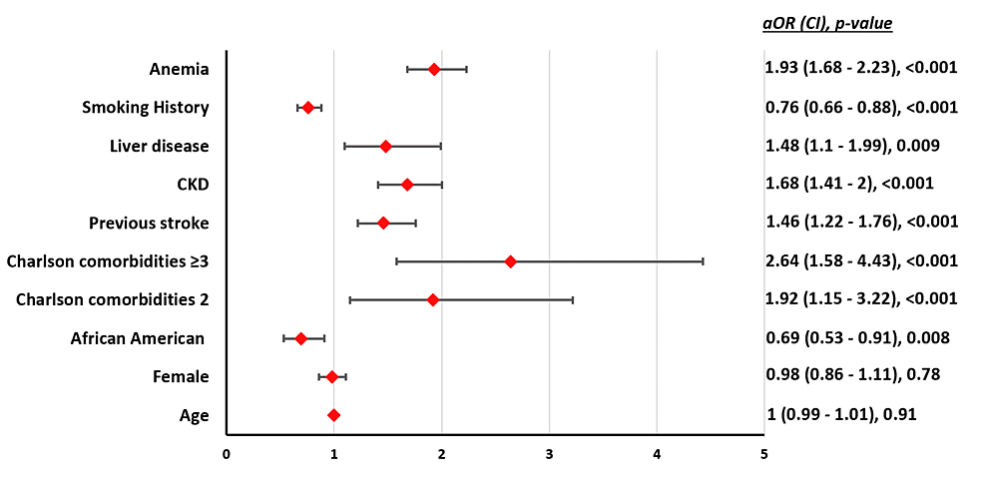
Independent factors associated with MAEs in patients aged <80 years undergoing TAVR. CKD: chronic kidney disease; aOR = adjusted odds ratio; CI = confidence interval; MAE: major adverse event; TAVR: transcatheter aortic valve replacement

The mean age for patients with and without MAEs was 71.2 (SE = ±0.19) vs 71.4 (SE = ±0.13), respectively (p < 0.001). The proportion of patients with comorbidities was significantly higher among those who experienced MAEs compared to those who did not (Table 3). Anemia (aOR = 1.93), liver disease (aOR = 1.48), CKD (aOR = 1.68), history of stroke (aOR = 1.46), and multiple comorbidities were independently associated with higher odds of MAEs. African American (aOR = 0.69) race was associated with lower odds of developing MAEs (Figure 4).

Comparison of TAVR patients with MAEs and age ≥80 years versus <80 years

Among patients with in-hospital MAEs, group B had higher comorbidities compared to group A (Charlson category ≥3 = 74.5 vs. 67%, p < 0.001). More patients in group A also belonged to zip codes with higher median incomes (p < 0.001). On multivariate analysis, patients in group A had higher in-hospital mortality compared to those in group B (5.7 vs. 4.3%, aOR = 1.58 [CI = 1.08-2.32], p = 0.01). The LOS was also shorter for those in group A (7.2 vs. 8.7 days, p = 0.03). There was no significant difference in the total hospital charges ($283,618 vs. $300,624, p = 0.17) between the two groups.

## Discussion

Our study shows that octogenarians undergoing TAVR had higher in-hospital mortality compared to those aged <80 years. There was no significant difference in the rates of MAEs between the two groups. Anemia, CKD, liver disease, and previous stroke were associated with higher odds of MAEs in both groups.

Irrespective of the indication for admission, studies have shown increased in-hospital complication rates associated with older age resulting in worse outcomes [12]. TAVR has been shown to be a relatively safe procedure even in nonagenarians [13]. Our study shows that age over 80 years is associated with 1.5 times higher odds of in-hospital mortality. These results are of particular interest, given previous studies on similar comparisons have shown conflicting data. Buellesfeld et al. in their study of 1,386 TAVR patients showed no difference in mortality among four age groups ranging from 40 to 99 years [14]. Havakuk et al. in their study comparing patients more than and less than 85 years of age reported no significant difference in in-hospital and 30-day mortality between the two groups but noticed higher mortality in the older group on follow-up [15]. Yamamoto et al. in their comparison of patients aged >90 to <90 showed a trend of higher mortality in the older groups albeit not statistically significant [16]. In their study of the TVT registry, Arsalan et al. showed higher in-hospital death was observed among nonagenarians (6.5% vs. 4.5%, p < 0.001) [17]. Considering that patients aged ≥80 years had a lower proportion of comorbidities in our study compared to their younger cohorts, further risk stratification models may be needed to assess the factors influencing mortality in older patients undergoing TAVR.

Our study identified that anemia was associated with higher odds of in-hospital MAE in all subgroups. TAVR patients with anemia ≥80 years old had marginally higher odds of MAE compared to those <80 years old (a0R = 2.12 vs. 1.93). Anemia has been reported to be present in 30% of patients with aortic stenosis [18]. Several studies have shown that anemia has been associated with worse short and long-term outcomes in patients undergoing TAVR [19-24]. A meta-analysis by Kanjanahattakij et al. showed increased long-term mortality but no change in short-term mortality [25].

Our data also showed that liver disease was associated with higher odds of MAEs in both groups. The older subgroup had lower prevalence but slightly higher odds (a0R = 1.48 vs. 1.57) of MAEs with liver disease. Previous studies have shown that liver disease is associated with higher mortality and morbidity in patients undergoing TAVR. A multicenter study by Tirado-Conte et al. showed that long-term non-cardiac mortality was higher in those with liver disease, especially those with Child-Pugh B and C cirrhosis, but in-hospital mortality (7%) was not affected by liver disease [26]. In their retrospective study of 640 patients, Wendt et al. showed that patients with liver cirrhosis undergoing TAVR had an in-hospital mortality rate of 36.4% [27]. The possible variation could be due to the difference in the severity of liver disease which cannot be assessed using the NIS database.

Our data show that TAVR patients <80 years old have a higher comorbidity burden compared to those ≥80 years old, except for stroke and PVD. The possible explanation for this could be the wide use of risk stratification tools for patients undergoing TAVR, namely, the Society of Thoracic Surgeons and EUROSCORE II models. These models place older patients with aortic stenosis and multiple comorbidities at higher risk of adverse outcomes, thereby resulting in reduced TAVR utilization in this population [28,29].

Pacemaker insertion is a common procedure-related complication of TAVR [30]. Despite the rapid technological advances in TAVR procedure, conduction abnormalities continue to be a significant complication requiring pacemaker insertion. Our study shows patients aged ≥80 had higher post-TAVR pacemaker insertion rates compared to those <80 years of age. Although not evaluated in this study, pre-existing conduction abnormalities and type of device have been known to be the strongest predictors of requiring a pacemaker. Increasing age is independently predictive of the need for a permanent pacemaker (PPM) after TAVR [30]. Because of cardiac remodeling and the higher presence of calcinosis older age is well known to be associated with conduction abnormalities, including bradyarrhythmia and tachyarrhythmia. This is likely the causative factor for higher PPM insertion rates in this population.

Our study has several limitations. First, data from the NIS is subject to biases associated with retrospective studies. Given that data are interpreted from NIS based on ICD codes, errors in coding may affect data accuracy. Additionally, due to the inherent design of NIS, long-term follow-up of outcomes is not possible. Because laboratory and pharmacological data were unavailable, utilization and comparison with STS and EUROSCORE were not feasible. Furthermore, comparing the rates of pacemaker insertion cannot be done because most studies report 30-day rates of pacemaker insertion rather than in-hospital rates. Finally, the NIS does not include information about the severity of the diagnosis at the time of admission. For example, the New York Heart Association stage of heart failure or stage of CKD could not be assessed.

## Conclusions

Overall, our study shows that in patients undergoing TAVR, the in-hospital mortality was slightly higher in patients ≥80 years old compared to those <80 years old. However, the rates of MAEs were not significantly different between the two groups. Further prospective studies are required to build risk stratification models for older patients who undergo TAVR.

## References

[1] Faggiano P, Antonini-Canterin F, Baldessin F, Lorusso R, D'Aloia A, Cas LD (2006). Epidemiology and cardiovascular risk factors of aortic stenosis. Cardiovasc Ultrasound.

[2] Osnabrugge RL, Mylotte D, Head SJ (2013). Aortic stenosis in the elderly: disease prevalence and number of candidates for transcatheter aortic valve replacement: a meta-analysis and modeling study. J Am Coll Cardiol.

[3] Danielsen R, Aspelund T, Harris TB, Gudnason V (2014). The prevalence of aortic stenosis in the elderly in Iceland and predictions for the coming decades: the AGES-Reykjavík study. Int J Cardiol.

[4] Smith CR, Leon MB, Mack MJ (2011). Transcatheter versus surgical aortic-valve replacement in high-risk patients. N Engl J Med.

[5] Leon MB, Smith CR, Mack M (2010). Transcatheter aortic-valve implantation for aortic stenosis in patients who cannot undergo surgery. N Engl J Med.

[6] Leon MB, Smith CR, Mack MJ (2016). Transcatheter or surgical aortic-valve replacement in intermediate-risk patients. N Engl J Med.

[7] Sheng SP, Strassle PD, Arora S (2019). In-hospital outcomes after transcatheter versus surgical aortic valve replacement in octogenarians. J Am Heart Assoc.

[8] Hirji SA, Ramirez-Del Val F, Kolkailah AA (2017). Outcomes of surgical and transcatheter aortic valve replacement in the octogenarians-surgery still the gold standard?. Ann Cardiothorac Surg.

[9] Yamamoto M, Mouillet G, Meguro K (2014). Clinical results of transcatheter aortic valve implantation in octogenarians and nonagenarians: insights from the FRANCE-2 registry. Ann Thorac Surg.

[10] Otto CM, Nishimura RA, Bonow RO (2021). 2020 ACC/AHA guideline for the management of patients with valvular heart disease: a report of the American College of Cardiology/American Heart Association Joint Committee on Clinical Practice Guidelines. Circulation.

[11] Sundararajan V, Quan H, Halfon P, Fushimi K, Luthi JC, Burnand B, Ghali WA (2007). Cross-national comparative performance of three versions of the ICD-10 Charlson index. Med Care.

[12] Flacker JM (2003). What is a geriatric syndrome anyway?. J Am Geriatr Soc.

[13] Mentias A, Saad M, Desai MY (2019). Temporal trends and clinical outcomes of transcatheter aortic valve replacement in nonagenarians. J Am Heart Assoc.

[14] Buellesfeld L, Gerckens U, Erbel R (2012). Age-stratified baseline and outcome characteristics of patients undergoing transcatheter aortic valve implantation: results from the German multicenter registry. J Invasive Cardiol.

[15] Havakuk O, Finkelstein A, Steinvil A (2014). Comparison of outcomes in patients ≤85 versus >85 years of age undergoing transcatheter aortic-valve implantation. Am J Cardiol.

[16] Yamamoto M, Meguro K, Mouillet G (2012). Comparison of effectiveness and safety of transcatheter aortic valve implantation in patients aged ≥90 years versus <90 years. Am J Cardiol.

[17] Arsalan M, Szerlip M, Vemulapalli S (2016). Should transcatheter aortic valve replacement be performed in nonagenarians?: Insights from the STS/ACC TVT registry. J Am Coll Cardiol.

[18] Tjahjadi C, Wee Y, Hay K, Tesar P, Clarke A, Walters DL, Bett N (2017). Heyde syndrome revisited: anaemia and aortic stenosis. Intern Med J.

[19] Arai T, Morice MC, O'Connor SA (2015). Impact of pre- and post-procedural anemia on the incidence of acute kidney injury and 1-year mortality in patients undergoing transcatheter aortic valve implantation (from the French Aortic National CoreValve and Edwards 2 [FRANCE 2] Registry). Catheter Cardiovasc Interv.

[20] DeLarochellière H, Urena M, Amat-Santos IJ (2015). Effect on outcomes and exercise performance of anemia in patients with aortic stenosis who underwent transcatheter aortic valve replacement. Am J Cardiol.

[21] Hellhammer K, Zeus T, Verde PE (2016). Red cell distribution width in anemic patients undergoing transcatheter aortic valve implantation. World J Cardiol.

[22] Nuis RJ, Sinning JM, Rodés-Cabau J (2013). Prevalence, factors associated with, and prognostic effects of preoperative anemia on short- and long-term mortality in patients undergoing transcatheter aortic valve implantation. Circ Cardiovasc Interv.

[23] Rheude T, Pellegrini C, Michel J (2017). Prognostic impact of anemia and iron-deficiency anemia in a contemporary cohort of patients undergoing transcatheter aortic valve implantation. Int J Cardiol.

[24] Seiffert M, Conradi L, Gutwein A (2017). Baseline anemia and its impact on midterm outcome after transcatheter aortic valve implantation. Catheter Cardiovasc Interv.

[25] Kanjanahattakij N, Rattanawong P, Krishnamoorthy P (2019). Anaemia and mortality in patients with transcatheter aortic valve replacement: a systematic review and meta-analysis. Acta Cardiol.

[26] Tirado-Conte G, Rodés-Cabau J, Rodríguez-Olivares R (2018). Clinical outcomes and prognosis markers of patients with liver disease undergoing transcatheter aortic valve replacement: a propensity score-matched analysis. Circ Cardiovasc Interv.

[27] Wendt D, Kahlert P, Canbay A (2017). Impact of liver indicators on clinical outcome in patients undergoing transcatheter aortic valve implantation. Ann Thorac Surg.

[28] Society of Thoracic Surgeons (2022). STS short-term risk calculator. The annals of thoracic surgery. https://www.sts.org/resources/risk-calculator.

[29] Group ES. euroSCORE II INTERACTIVE CALCULATOR (2022). EuroSCORE II interactive calculator. http://www.euroscore.org/calc.html.

[30] Huang HD, Mansour M (2020). Pacemaker implantation after transcatheter aortic valve replacement: a necessary evil perhaps but are we making progress?. J Am Heart Assoc.

